# ChatGPT’s Performance in Spinal Metastasis Cases—Can We Discuss Our Complex Cases with ChatGPT?

**DOI:** 10.3390/jcm13247864

**Published:** 2024-12-23

**Authors:** Stephan Heisinger, Stephan N. Salzmann, Wolfgang Senker, Stefan Aspalter, Johannes Oberndorfer, Michael P. Matzner, Martin N. Stienen, Stefan Motov, Dominikus Huber, Josef Georg Grohs

**Affiliations:** 1 Department of Orthopedics and Trauma Surgery, Medical University of Vienna, 1090 Vienna, Austria; stephan.heisinger@meduniwien.ac.at (S.H.);; 2 Department of Neurosurgery, Kepler University Hospital, 4020 Linz, Austriastefan.aspalter@kepleruniklinikum.at (S.A.);; 3 Spine Center of Eastern Switzerland & Department of Neurosurgery, Kantonsspital St. Gallen, Medical School of St. Gallen, University of St.Gallen, 9000 St. Gallen, Switzerland; 4 Division of Oncology, Department of Medicine I, Medical University of Vienna, 1090 Vienna, Austria

**Keywords:** ChatGPT, spine, decision making, spinal metastasis, spine surgery

## Abstract

**Background:** The integration of artificial intelligence (AI), particularly large language models (LLMs) like ChatGPT-4, is transforming healthcare. ChatGPT’s potential to assist in decision-making for complex cases, such as spinal metastasis treatment, is promising but widely untested. Especially in cancer patients who develop spinal metastases, precise and personalized treatment is essential. This study examines ChatGPT-4’s performance in treatment planning for spinal metastasis cases compared to experienced spine surgeons. **Materials and Methods:** Five spine metastasis cases were randomly selected from recent literature. Consequently, five spine surgeons and ChatGPT-4 were tasked with providing treatment recommendations for each case in a standardized manner. Responses were analyzed for frequency distribution, agreement, and subjective rater opinions. **Results:** ChatGPT’s treatment recommendations aligned with the majority of human raters in 73% of treatment choices, with moderate to substantial agreement on systemic therapy, pain management, and supportive care. However, ChatGPT’s recommendations tended towards generalized statements, with raters noting its generalized answers. Agreement among raters improved in sensitivity analyses excluding ChatGPT, particularly for controversial areas like surgical intervention and palliative care. **Conclusions:** ChatGPT shows potential in aligning with experienced surgeons on certain treatment aspects of spinal metastasis. However, its generalized approach highlights limitations, suggesting that training with specific clinical guidelines could potentially enhance its utility in complex case management. Further studies are necessary to refine AI applications in personalized healthcare decision-making.

## 1. Introduction

Artificial intelligence (AI) is gradually changing almost every aspect of our lives and specifically healthcare [1,2]. AI is designed to carry out tasks that require human intellect, learning, complex problem solving, and decision making [3]. The field of AI is constantly evolving and has exponentially progressed within the last few years, with its evolution currently peaking in commonly accessible AI-driven chatbots [2,3,4]. Based on natural language processing (NLP) and machine learning (ML), large language models (LLMs) are capable of leveraging complex neural network architectures [5,6]. As reviewed by Pressman et al., these AI-based LLMs are trained on vast amounts of text data and consequently develop the ability to comprehend and generate high quality texts [5,7]. As the most commonly known LLM, “ChatGPT” has created an immense impact since its’ launch in 2022 by the AI lab OpenAI [1,8]. ChatGPT uses generative pre-trained transfer (GPT), which resembles an NLP model and has been trained to generate comprehensive and contextual responses to a great variety of questions and topics [1,9,10]. ChatGPT has the potential to revolutionize patient care in orthopedics, considering its ability to detect patterns and provide insights to further enhance decision-making and improve surgical precision [1,11]. Although ChatGPT has a promising potential to support orthopedic surgeons, patients, and medical students alike, there are ethical concerns and certain limitations in regard to the responses, which may result from biases and limitations of the training data [1,11,12,13,14,15]. Moreover, ChatGPT’s involvement in healthcare raises ethical concerns in regard to accountability and responsibility [1,16,17,18,19]. Nevertheless, considering ChatGPT’s ability to detect patterns and process vast amounts of information, depending on its training data, it has the potential to provide significant support in decision-making [1,3,20].

In spine surgery, specifically regarding the management of spinal metastasis, decision making is highly complex considering the multifaceted nature of the disease and requires individualized and multimodal treatment approaches [21]. Taking into consideration that it is estimated that up to 70% of cancer patients develop a spinal lesion and the spine is the most common site for bony metastasis, this further highlights the relevance of establishing a personalized treatment regimen based on the most recent literature [22,23,24,25]. Considering the advancements in oncology, radiotherapy, and spinal surgery, decision finding is becoming more and more complex and multidisciplinary, and moreover, balancing survival and quality of life is a crucial yet still controversial aspect in this field [22].

Given the complexity of treating metastatic spine disease and ChatGPT’s aforementioned qualities, we set out to explore its potential to support decision-making and establish a treatment plan for spinal oncology.

## 2. Materials and Methods

**Figure 1:**
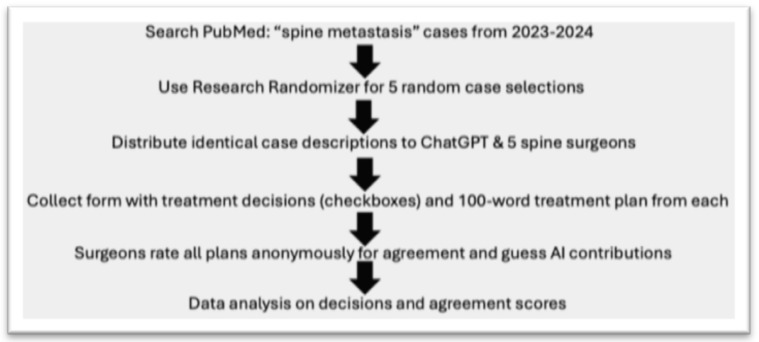
Flow chart of the study design.

The objective of this study was to compare the decision-making capability of ChatGPT 4 with that of five human spine surgeons regarding therapeutic decisions in different cases of patients with spinal metastasis. Figure 1 summarizes the study design.

For this purpose, a PubMed search was conducted searching for recent case reports of the years 2023–2024 with the search words “spine metastasis”. This search was conducted in May of 2024 and resulted in a total of 114 case reports in the currently existing literature. Subsequently, a free software program (Research Randomizer (Version 4.0)) was utilized to select five random case reports out of these 114 to ensure diversity. The 5 randomly selected case reports are shown in Table 1.

**Figure 2:**
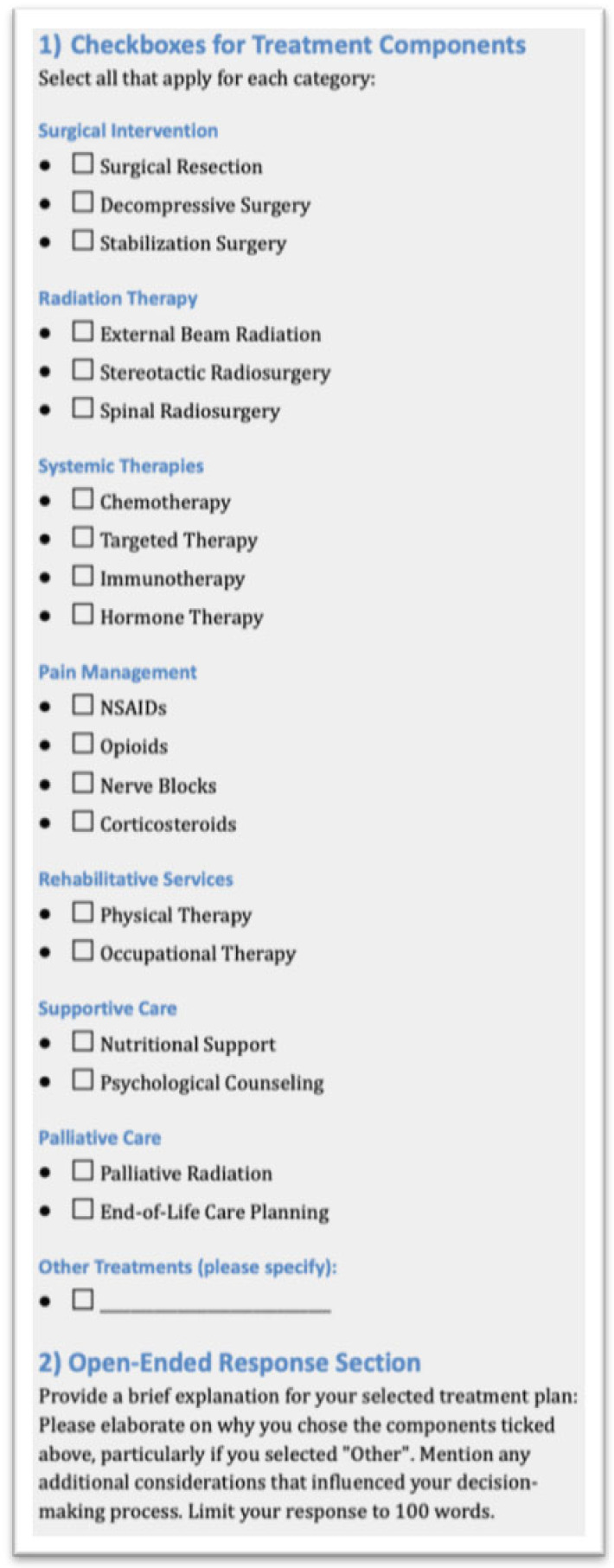
Response form used to collect data from five spine surgeons and ChatGPT.

Identical case descriptions were prepared omitting any information on the performed treatment used by the authors of the original case report. Identical case descriptions were then provided to 5 spine surgeons from 3 different spine centers, 3 neurosurgeons and 2 orthopedic surgeons with 12.3 ± 8.84 years of experience in spine surgery, as well as to the paid version of ChatGPT 4. A two-step process was then used to collect the data. In a first step, the surgeons and ChatGPT completed a response form including checkboxes for treatment recommendations from predefined categories such as Surgical Intervention, Radiation Therapy, Systemic Therapies etc. In addition, the participants completed an open-ended response section elaborating on their choices within a 100-word limit. The response form is depicted in Figure 2.

In a second step, all six response forms (five spine surgeons and ChatGPT) were anonymously provided to the five human raters (spine surgeons) to rate their agreement with the treatment plan and guess the contribution of ChatGPT. Additionally, statements of the five human raters on why they guessed a specific rater to be ChatGPT were collected.

From an ethical point of view, only publicly available anonymized case reports were used for this study. Therefore, institutional review board approval was not required.

### Statistics

All data were stored and processed for analysis in MS Excel ((Microsoft Corporation, 2018. Microsoft Excel, available at: https://office.microsoft.com/excel, accessed on 1 July 2024) and then analyzed in R (R Core Team (2021). R: A language and environment for statistical computing available at: https://www.R-project.org/, accessed on 1 July 2024). For the analysis of binary answers, the median and quartiles were used, and Fisher’s exact test was used for frequency distribution. Rater agreement was measured via Fleiss’ Kappa and appropriate levels of agreement applied [31,32].

## 3. Results

**Figure 3:**
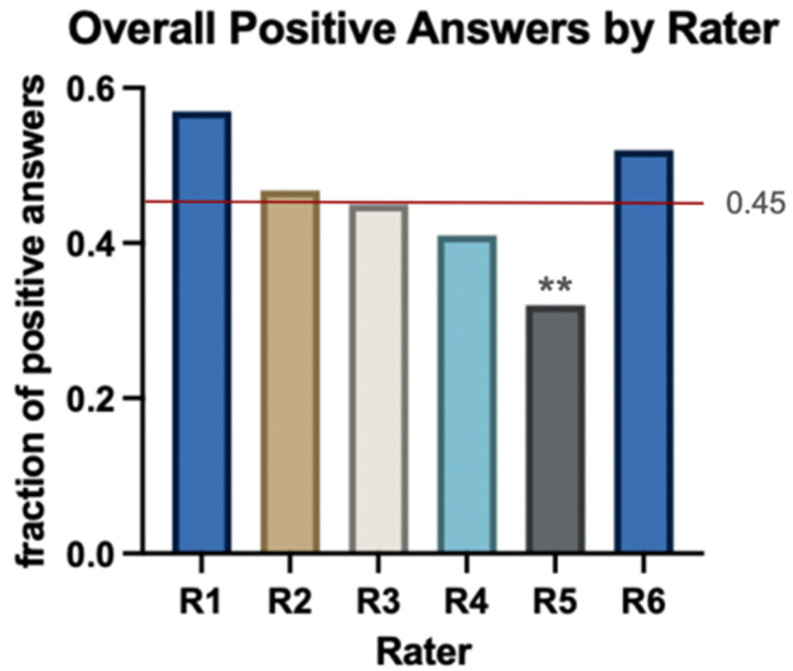
Relative frequencies of overall positive answers to the 100 questions asked by each rater. Rater 1 to 5 were human, rater 6 was ChatGPT. Fisher’s exact test showed significant difference in distribution at *p* < 0.001, with a Bonferroni-corrected post hoc analysis determining rater five to have given significantly fewer “yes” answers (**). ChatGPT has 52% positive answers above the average of 45.6%.

A total of 20 questions for each of the five cases were answered by each rater. There were 23 missing values for one rater (Rater 2), 7 occurring in case 1 and 16 in case 2. All other raters, including ChatGPT, gave valid answers to all 100 questions. On average, 53.1% of the 100 questions were answered with “yes”. Rater 5 answered significantly fewer times with “yes” (32%, Fisher’s Exact Test for difference in distributions *p* < 0.001). ChatGPT’s number of “yes” answers and, therefore, therapy-recommendations (n = 52; 52%) was above the average (45.6%, Figure 3).

**Figure 4:**
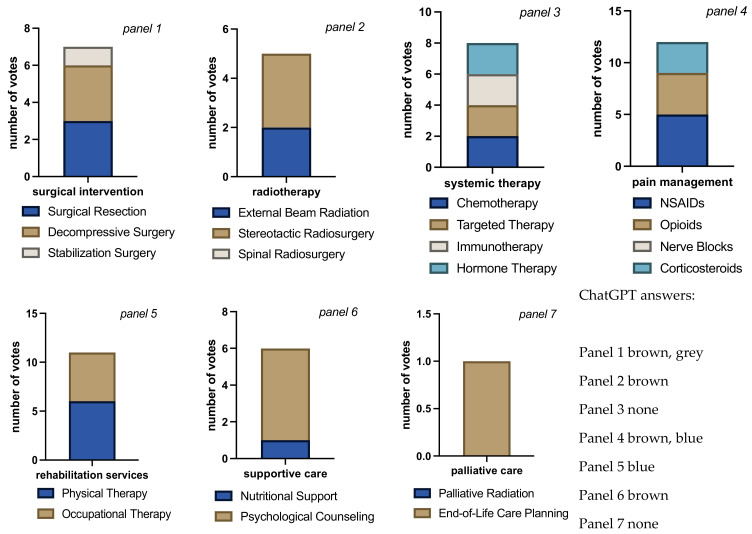
Bar charts for total number of votes per question block for each intervention/therapy decision in case 1. Bar chart length shows the sum of cast votes, and each color block within the bar shows the absolute number of votes for each available option. Each rater could choose none, all, or any combination of options in each question block. The multiple colors in the bars indicate that raters considered more than one answer appropriate (panels 1 to 6). The different sizes of color-blocks in each bar indicate raters’ propensity towards a certain answer (e.g., panel 6, raters favoring psychological counseling over nutritional support). Evenly distributed colors could either mean total agreement that all chosen answers were correct or total disagreement, each rater choosing a different answer (panel 3, in this case disagreement). The mono-colored bar chart in panel 7 indicates total rater agreement on end-of-life care planning with no votes for palliative radiation. Overall rater agreement was the lowest on this case compared to all other cases (Fleiss’ Kappa = 0.42, “moderate agreement”, *p* < 0.001).

Case-by-case, the distribution of answers differed significantly (Fisher’s exact test *p* < 0.001) reflecting the different situations presented in each case. For case 1, the vote was tied for either surgical resection or decompressive surgery (3 each), with ChatGPT voting for decompression and casting the only vote for stabilization (Figure 4). No rater considered spinal radiation, 3 raters, including ChatGPT, voted for stereotactic and 2 for external beam radiation. In terms of systemic therapy, 2 raters voted for all available options, and for 3 raters, including ChatGPT, no answer was correct, and 1 rater gave no answers, leaving the vote tied for chemotherapy, immunotherapy, hormone therapy, and targeted therapy. This situation may also indicate an issue with this particular question and raise doubt about the validity of the inferred result. All but one rater opted for pain management with NSAIDs, 4 raters, including ChatGPT, suggested WHO grade II pain medication, and 3 human raters suggested additional corticosteroids. No rater voted for nerve block. All raters agreed on the indication for physical therapy, and 4 raters also suggested occupational therapy. Only 1 (human) rater opted for nutritional support. All agreed on the necessity of psychological counseling as well as end-of-life care planning, with no votes for palliative radiation therapy. In summary, raters in case 1 would suggest surgical decompression or resection and stereotactic radiation. There is no clear strategy regarding systemic therapy, but there is a consensus for NSAIDs in pain management with possibly opioid or corticosteroid add-on. Physical therapy, along with occupational therapy, is recommended, and there is clear support for psychological counseling and end-of-life care planning.

**Figure 5:**
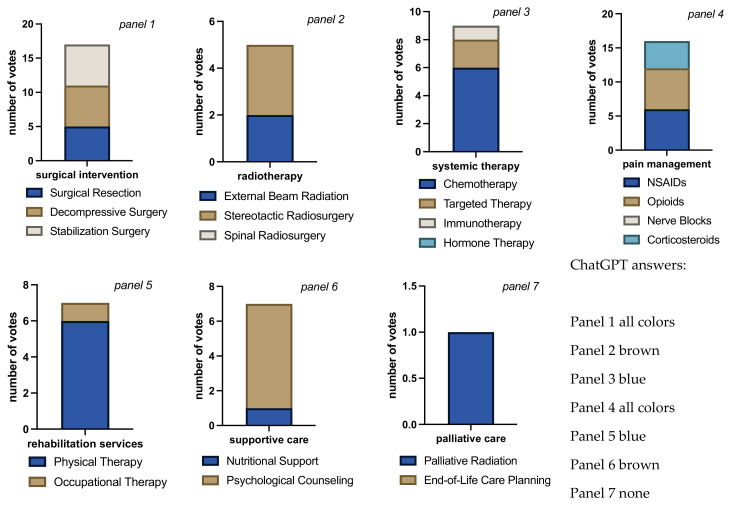
Bar charts for total number of votes per question block for each intervention/therapy decision in case 2. Bar chart length shows the sum of cast votes, and each color block within the bar shows the absolute number of votes for each available option. Each rater could choose none, all, or any combination of options in each question block. The multiple colors in the bars indicate that raters considered more than one answer appropriate (panels 1 to 6). The different sizes of color-blocks in each bar indicate raters’ propensity towards a certain answer (e.g., panel 6, raters favoring psychological counseling over nutritional support). Evenly distributed colors could either mean total agreement that all chosen answers were correct or total disagreement, each rater choosing a different answer (panel 4, in this case agreement). The mono-colored bar chart in panel 7 indicates only 1 vote cast with 5 voters seeing no need for palliative care and 1 human voter opting for palliative radiation. Overall rater agreement was the highest on this case compared to all other cases (Fleiss’ Kappa = 0.60, “substantial agreement”, *p* < 0.001).

In Case 2, all raters opted for decompressive stabilization surgery, and all but one human rater also suggested resection (Figure 5). Three raters, including ChatGPT, opted for stereotactic radiosurgery, 2 for external radiation, and none considered spinal radiosurgery. All raters agreed on the indication to chemotherapy, 2 human raters also suggested targeted therapy, and 1 human rater voted for immunotherapy. Hormone therapy was not an option for any rater. The combination of NSAIDs and opioids was agreed upon by all raters, 4 of whom, including ChatGPT, also saw an indication for corticosteroids. Nerve block received zero votes. All 6 voters agreed on physical therapy and psychological counseling, and 1 human voter also opted for nutritional support and occupational therapy. None of the raters voted for end-of-life care planning, and palliative radiation received only 1 (human) vote. In summary, raters in case 2 agreed upon decompression and stabilization and possibly resection followed by chemotherapy. WHO II pain management, along with physical therapy and psychological counseling, were the consensus in this case. Palliative care is not a majority recommendation.

**Figure 6:**
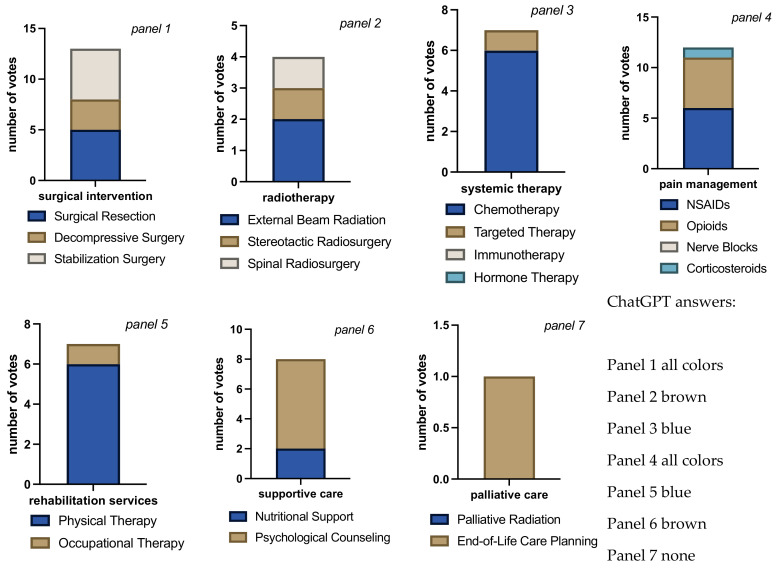
Bar charts for total number of votes per question block for each intervention/therapy decision in case 3. Bar chart length shows the sum of cast votes, and each color block within the bar shows the absolute number of votes for each available option. Each rater could choose none, all, or any combination of options in each question block. The multiple colors in the bars indicate that raters considered more than one answer appropriate (panels 1 to 6). The different sizes of color-blocks in each bar indicate raters’ propensity towards a certain answer (e.g., panels 3, 5, and 6: raters favoring chemotherapy, physical therapy, and psychological counseling, respectively, over answer alternatives). Evenly distributed colors could either mean total agreement that all chosen answers were correct or total disagreement, each rater choosing a different answer (panel 1, in this case agreement). The mono-colored bar chart in panel 7 indicates only 1 vote cast with 5 voters seeing no need for palliative care and 1 human voter opting for end-of-live care planning. Overall rater agreement was high on this case compared to all other cases (Fleiss’ Kappa = 0.60, *p* < 0.001) and highest among all cases if ChatGPT was left out of the calculation (Fleiss’ Kappa = 0.73, “substantial agreement”, *p* < 0.001).

Each 5 votes were cast for resection and stabilization surgery in case 3 (Figure 6). ChatGPT and 2 human raters also voted for decompressive surgery. External beam radiation received 2 votes, and 1 vote was cast for stereotactic radiation and spinal radiosurgery, the latter by the AI. All raters saw an indication for chemotherapy, only ChatGPT also suggested targeted therapy. No vote was cast for hormone or immuno-therapy. NSAIDs received all 6 votes, opioids 5 votes, and only ChatGPT opted for the use of corticosteroids. Again, physical therapy and psychological counseling were agreed upon by all voters, while occupational therapy received 1 vote and nutritional support 2 votes. There was only 1 (human) vote for end-of-life care planning), all other raters voted for no palliative care. In summary, raters see in case 3 the indication for resection and stabilization as well as chemotherapy. Pain management with NSAIDs and opioids is also recommended, along with physical therapy and psychological counseling. Palliative care is not a majority recommendation in this case.

**Figure 7:**
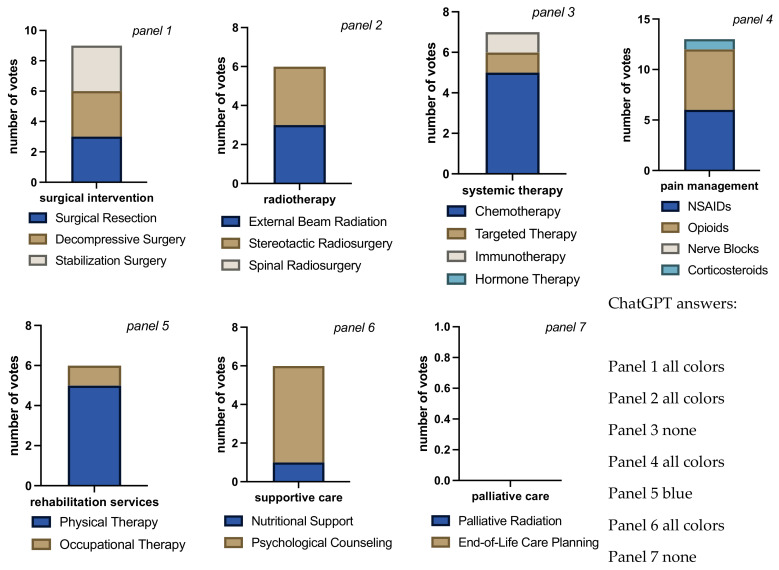
Bar charts for total number of votes per question block for each intervention/therapy decision in case 4. Bar chart length shows the sum of cast votes and each color block within the bar the absolute number of votes for each available option. Each rater could choose none, all, or any combination of options in each question block. The multiple colors in the bars indicate that raters considered more than one answer appropriate (panels 1 to 6). The different sizes of color-blocks in each bar indicate raters’ propensity towards a certain answer (e.g., panels 5 and 6: raters favoring physical therapy and psychological counseling, respectively, over answer alternatives). Evenly distributed colors could either mean total agreement that all chosen answers were correct or total disagreement, each rater choosing a different answer (panel 1, in this case disagreement). No bar in panel 7 indicates that no rater is seeing a need for palliative care. Overall rater agreement was high on this case compared to all other cases (Fleiss’ Kappa = 0.58, “moderate agreement”, *p* < 0.001) and even higher if ChatGPT was left out of the calculation (Fleiss’ Kappa = 0.66, “substantial agreement”, *p* < 0.001).

In case 4, surgical resection, decompressive, and stabilization surgery each received 3 votes, with ChatGPT choosing all three options as well as stereotactical radiosurgery (Figure 7). The latter and external beam radiation were each chosen by 3 raters, spinal radiosurgery received no votes. All raters who cast votes agreed on chemotherapy, 1 (human) rater also considered immunotherapy and targeted therapy. Hormone therapy received no votes, and 1 rater cast no votes on this question. All 6 raters agreed on a combination of NSAIDs and opioids for pain management, and ChatGPT also suggested the use of corticosteroids. Similar to the previous cases, physiotherapy and psychological counseling each received 5 votes (including ChatGPT while occupational therapy and nutritional support were chosen once each. There was unanimous consent that no palliative care was necessary. In summary, no clear strategy could be derived for surgical intervention with ChatGPT making no difference by choosing all three options. There is also no clear majority for either of the radiation options, except spinal radiosurgery is not being indicated. There is a clear vote for chemotherapy and a combination of NSAIDs with opioids, physiotherapy, and psychological counseling, as well as no necessity for palliative care.

**Figure 8:**
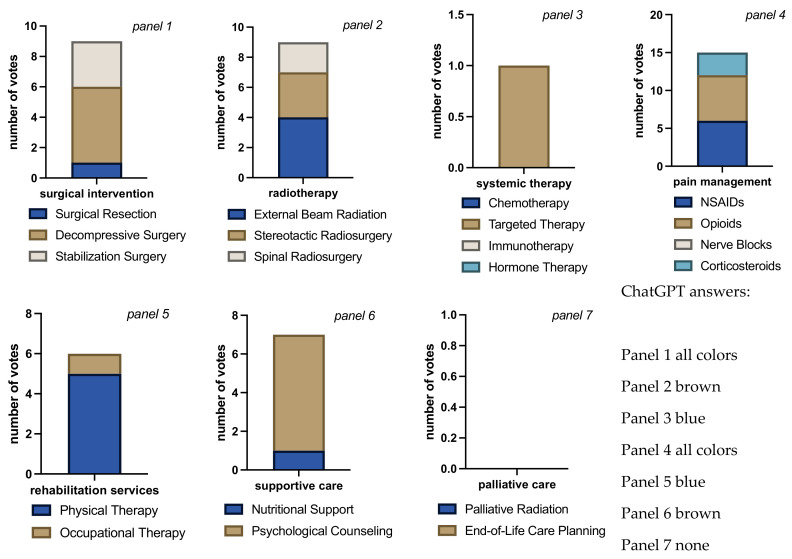
Bar charts for total number of votes per question block for each intervention/therapy decision in case 5. Bar chart length shows the sum of cast votes, and each color block within the bar shows the absolute number of votes for each available option. Each rater could choose none, all, or any combination of options in each question block. The multiple colors in the bars indicate that raters considered more than one answer appropriate (e.g., panel 1). The different sizes of color-blocks in each bar indicate raters’ propensity towards a certain answer (e.g., panels 5 and 6: raters favoring physical therapy and psychological counseling, respectively, over answer alternatives). Evenly distributed colors could either mean total agreement that all chosen answers were correct or total disagreement, each rater choosing a different answer (panel 4, in this case agreement). No bar in panel 7 indicates that no rater is seeing a need for palliative care. Overall rater agreement was low on this case compared to all other cases (Fleiss’ Kappa = 0.48, “moderate agreement”, *p* < 0.001) and lowest among all cases if ChatGPT was left out of the calculation (Fleiss’ Kappa = 0.40, “moderate agreement”, *p* < 0.001).

For case 5, 5 raters opted for decompression surgery, 3 voted for stabilization, and ChatGPT was the only rater to suggest surgical resection (Figure 8). External beam radiation was recommended by 4 raters, stereotactic radiosurgery by 3, and spinal radiosurgery by 2 raters, with ChatGPT voting for the latter two options. No rater chose chemotherapy, immunotherapy, or hormone therapy, and only 1 (human) rater recommended targeted therapy. All raters voted unanimously for the combination of NSAIDs and opioids, 3 raters, including ChatGPT, suggested the addon of corticosteroids, and none voted for nerve blocks. Physical therapy (5 votes) and occupational therapy (1 vote) were recommended alongside psychological counseling (6 votes) and nutritional support (1 vote). All raters agreed on not recommending palliative care. In summary, in case 5, raters recommend decompression and no systemic therapy. There is no clear consensus regarding radiation but a clear majority for NSAIDs with opioids and no palliative care. Similar to the other cases, physical therapy and psychological counseling are favored by a majority of voters.

**Figure 9:**
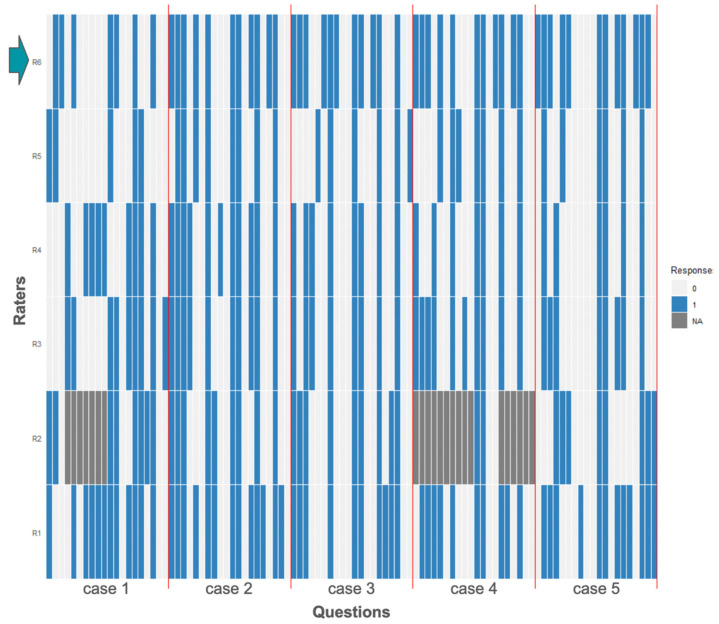
Answer Heatmap for each of 5 cases (separated by red vertical lines) shows the same 20 questions (x-axis, each question represented by 1 column) were answered by 6 raters (y-axis, rows) with either yes (blue color) or no light (grey color). Unanswered questions are indicated by dark grey color. A total of 20 questions were answered the same by all 6 raters, indicated by a column that is either all blue or all light grey. ChatGPT as rater 6 (row 1) is indicated by a cyan arrow.

Overall, there was moderate case- and question-agnostic rater agreement measured by Fleiss’ Kappa (Fleiss Kappa 0.548, *p* < 0.001). Out of 100 questions, 37 (37%) were answered unanimously, of which 17 were positive agreements and 20 were unequivocally answered with “no” (Figure 9). For 85% of questions, there was a majority for either “yes” or “no” (34 answers 5:1, 14 answers 4:2), and 15 answers were tied.

When Rater 6 (=ChatGPT) was excluded in sensitivity analyses, 47 out of 100 questions were answered unanimously with 29 positive and 18 negative agreements. A total of 36 were voted 4:1, and 17 3:2. Fleiss’ Kappa without ChatGPT as a rater was higher, at 0.60 (*p* < 0.001, “substantial agreement”). In 90% of the questions, there was at least 1 rater agreeing with ChatGPT. In 10 questions, ChatGPT had no support, with 9 “yes” answers and 1 “no” answer for which all other raters voted for the opposite. In 43 negative and 30 positive answers, ChatGPT voted for the majority answer, which translates to 73% of questions where the majority of experts agree with the ChatGPT solution.

**Figure 10:**
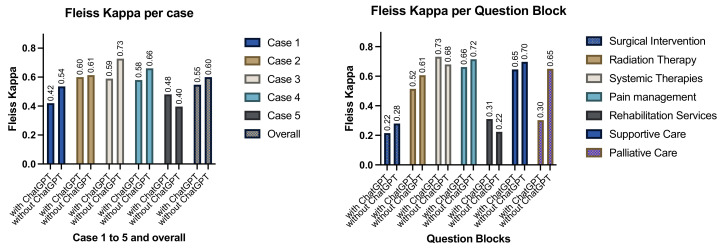
**Left Panel** results of a case-by-case Fleiss’ Kappa estimation (y-axis, 0.2 to 0.39 “fair”, 0.4 to 0.59 “moderate”, 0.6 and above “substantial agreement) grouped by cases and overall result (x-axis, each case and the overall estimation have separate colors. The left bar in each color is an estimation with ChatGPT included, the right bar is the result without ChatGPT. Rater Agreement in cases 1 to 4, as well as overall increases without ChatGPT contributing. **Right Panel** results of Fleiss’ Kappa calculation by question block. Each block (x-axis) is in the same color, the left bar in each color represents the result with ChatGPT considered, and the right bar without the AI. Controversial questions revolved around surgical intervention, rehabilitation services, and palliative care. Except for rehabilitation, all question blocks profited from removing ChatGPT as rater.

In a case-by-case analysis, rater agreement differed from overall agreement, reflecting the heterogeneity of case studies. While the raters showed moderate to substantial agreement in cases 2, 3, and 4 (Fleiss Kappa 0.6, 0.59, 0.58 with *p*-values <0.001), agreement in case one and case 5 was fair to moderate. When ChatGPT was removed from the calculation, the impact on levels of agreement was different for each case. While cases 1, 3, and 4 experienced significant improvement in their Fleiss’ Kappa values, case 2 changed only marginally, and case 5 even got worse (Figure 10, left panel). In total, with or without ChatGPT, cases 1 and 5 remained relatively controversial, while cases 2 to 4 showed moderately high levels of agreement.

When grouped into the 7 question blocks (surgical intervention, radiation therapy, systemic therapy, pain management, rehabilitation services, supportive care, and palliative care), rater agreement differed greatly. There was controversy regarding surgical intervention (Fleiss’ Kappa 0.22, *p* = 0.028, “fair agreement”), rehabilitation services (Fleiss’ Kappa 0.31, *p* = 0.048, “fair agreement”), and palliative care (Fleiss’ Kappa 0.3, *p* = 0.033, “fair agreement”). Regarding radiation therapy, moderate agreement (Fleiss Kappa 0.52, *p* < 0.001) was estimated. Raters substantially agreed on choice of systemic therapy (Fleiss’ Kappa 0.73, *p* < 0.001), pain management (Fleiss’ Kappa 0.66, *p* < 0.001), and supportive care (Fleiss’ Kappa 0.65, *p* < 0.001). When corrected for multiple testing, only the latter 3 remain statistically significant at an alpha of 1%. Without ChatGPT in the calculation, there is substantial, statistically significant agreement regarding radiation therapy, systemic therapy, pain management, supportive care, and palliative care (Figure 10, right panel, right bars). In the already controversial surgical therapy block, there is not much change, and rehabilitation services even worsen.

In a second step, each human rater gave their personal opinion on each other rater’s overall answers, including ChatGPT, on a scale of 0 (complete disagreement) to 10 (full approval). Median approval was 7, with ChatGPT receiving a median approval of 7. Human raters were above or below the approval rating of 7 but not farther away than 1.5 points. ChatGPT was guessed right to be rated 6 by 4 out of 5 human raters. One rater guessed rater 5 to be ChatGPT. In their statement on why they guessed a rater to be ChatGPT, all human raters gave similar answers (Table 2), with “generalized statements” being a common denominator in all 5 statements with “language” being only implicitly mentioned.

## 4. Discussion

Recent studies have explored the potential of ChatGPT in spine surgery, demonstrating its ability to counsel patients, generate novel systematic review ideas with 68% accuracy, and provide recommendations for thromboembolic prophylaxis with varying accuracy between versions 3.5 and 4.0 [33,34,35]. ChatGPT showed promise in answering patient-focused questions about lumbar and cervical spine surgery, albeit with limitations in accuracy and readability [35,36]. Its ability to pass board-examinations, including the topic of spine surgery, has been demonstrated [37]. The model’s performance in generating clinical guidelines for antibiotic prophylaxis improved from GPT-3.5 to GPT-4.0 [38]. However, ChatGPT fell short in diagnosing and managing spinal pathologies compared to experienced surgeons [39]. The technology also showed potential in automating billing for spine surgery [34]. While AI/ML applications in spine surgery have shown promise, their integration into clinical practice requires caution and further development [40]. Although the application of ChatGPT has been evaluated for various tasks, its ability to establish treatment plans for complex spinal cases, more specifically spinal metastasis, has not been investigated yet. Interestingly, we found moderate overall case- and question-agnostic agreement, with 37% of questions being answered unanimously, further increasing to 47% if ChatGPT is excluded. However, we also observed that in 90% of the questions, at least one human rater agreed with ChatGPT, which may be attributed to the heterogeneity of the selected cases and varying approaches. More interestingly, there was moderate to substantial agreement between raters for cases 2,3, and 4, while cases 1 and 5 remained controversial, even when excluding ChatGPT, which also reflects the heterogeneity of case reports but also hints that ChatGPT generally fits in with the human raters and gives valid answers. Furthermore, we observed that there was controversy regarding surgical intervention, rehabilitation services, and palliative care, with only fair agreement among the raters, which we had expected, especially in regard to surgical interventions, since the rater’s choice may rather depend on his/her subjective assessment and potentially past experiences, etc., than on objective and reproducible assessments. Overall our results are in accordance with the findings by Chalchoub et al., however, we found that ChatGPT did fairly well in cases that could be regarded as more straight-forward and was mostly in-line with the other raters [39]. Interestingly, there was substantial agreement on systemic therapy, which may be due to the better availability of guidelines for systemic treatment. Surprisingly, all but one human rater correctly identified ChatGPT’s personal opinion, and when asked for the reason for their choice, all five raters predominantly mentioned the generalized answers and only implicitly the use of English.

When implementing AI and, more specifically, ChatGPT in clinical decision finding certain ethical considerations are raised and need to be further investigated and moreover considered [5]. As reviewed by Pressman et al., data protection and safety is are crucial aspects to avoid potential misuse of medical data or even identity theft and to maintain privacy and safeguard data security [5,41,42,43]. ChatGPT has the potential to improve various aspects in healthcare since it is easily accessible for patients and can promptly provide them with medical advice and assistance, thereby resembling a cost-effective alternative to in-person consultation, highlighting the socioeconomic aspect of AI applications [44,45,46]. Given its ability to converse in multiple languages, it can also provide non-native speakers with comprehensive information and tailored responses to patients based on their medical history, etc. [44,45,47]. However, there are various limitations, such as limited empathy, incapability to perform physical examinations, potentially lack of clinical judgment and experience to address complex medical conditions, etc. [44,46,48,49,50]. Another major pitfall may be that ChatGPT may have limited access to potentially crucial information to provide adequate responses [44,45,46,51]. Furthermore, since ChatGPT learns from large datasets that may contain biases reflecting the respective society’s views, it may also be prone to producing, e.g., discriminatory responses and consequently requires close monitoring in this regard [44,48,52,53]. As reviewed by Souza et al., maintaining transparency regarding ChatGPT’s limitations is crucial, and patients need to be aware that it is not a substitute for professional advice [44,54]. Apart from these aspects, legal concerns are raised in regard to legal responsibility and accountability for potentially harmful advice given by AI applications, consequently highlighting the urgent need for distinct laws and regulations [55]. Moreover, LLMs have been found to have the tendency to produce misinformation via hallucination, referring to an LLM generating seemingly plausible information that actually is not based on existing data [56,57,58]. Given the incredible pace at which this field is evolving, addressing ethical and legal concerns as well as concerns regarding privacy and data safety is crucial to ensure patient safety [44,55,56].

This study is not without limitations. The generalizability of our results might be limited due to the selection of five random case reports from the literature of the last two years. Therefore, the variety and complexity of the cases might not represent the full spectrum of encountered cases in daily clinical practice. Furthermore, since image processing is still limited by large language models, only text information was provided to AI and the raters. Additionally, although important parts of the surgical decision-making process, physical examination and patient preference could not be included in the current study. Further, it is unknown to the authors which training data and information were accessed by the AI as a basis for its decisions. Providing the LLM with specific guidelines as a basis and prompting it to use the uploaded guidelines for the decisions might be an interesting approach in the future. In addition, repeated queries from independent ChatGPT accounts were not performed. From the author’s experience, however, the results received after providing identical case descriptions from various ChatGPT accounts were not 100% consistent. Lastly, in terms of data collection, the received answers might have been restricted by the use of checkboxes and a 100-word limit for the open-ended questions. We considered translating the information on the primary treatment plan of the case report to another “virtual” observer, however, we dismissed the idea since validation of the resulting answers reflecting the given information would be questionable. Another limitation of our study is the heterogeneity of observers regarding their experience and training, as well as the limited number of observers, which we aim to address in future studies. Furthermore, the performance of other AI applications compared to ChatGPT needs to be investigated in future studies.

## 5. Conclusions

Our results suggest that ChatGPT has the potential to support future spine surgeons in decision-making but currently it adheres to generalized answers. A major pitfall in this regard is that we have no conclusive information on ChatGPT’s current training we believe that further studies are required in which the chatbot is pretrained with landmark publications and accepted clinical guidelines. Moreover, it should be tested and approved for its’ ability to implement medical information for use in daily patient care.

## Figures and Tables

**Table 1:** The 5 randomly selected case reports from the existing literature from 2023 to 2024 of patients with “spine metastasis”.

Case Number	Author and Publication Year	Title
1	Tan et al., 2023 [26]	Epithelioid Sarcoma of the Spine: A Review of Literature and Case Report.
2	Zhang et al., 2023 [27]	A Novel Technology for 3D-Printing Artificial Vertebral Bodies for Treating Lumbar Spine Adrenal Pheochromocytoma Metastases: A Case Report and Review of the Literature.
3	Mitra et al., 2023 [28]	Primary Osseous Leiomyosarcoma with Vertebral and Nodal Metastasis in a Young Woman: A Rare Case Report.
4	Agosti et al., 2023 [29]	Treatment strategy for vertebral metastases from anal squamous cell carcinoma: a comprehensive literature review and case report.
5	Zhou et al., 2024 [30]	Metastasis of Intracranial Meningioma to the Osseous Spine: Systematic Literature Review and Case Report.

**Table 2:** Statements of 5 human raters on why they guessed a specific rater to be ChatGPT. Four of the five raters guessed Rater 6 correctly to be AI. All raters fielded “general answers” as the main reason for their choice.

Anonymized statements of Raters 1 to 5	Short, redundant and similar answers to all questions with generally valid information.
	Took psychological counseling every time and when read head to head, each case with similar answers.
	General answers
	The answers are similar and kept general
	Generalised statements, well-written

## Data Availability

The data that support the findings of this study are available from the corresponding author, upon reasonable request.
